# Detection and Genetic Characterization of Adenovirus Type 14 Strain in Students with Influenza-Like Illness, New York, USA, 2014–2015

**DOI:** 10.3201/eid2307.161730

**Published:** 2017-07

**Authors:** Daryl M. Lamson, Adriana Kajon, Matthew Shudt, Gabriel Girouard, Kirsten St. George

**Affiliations:** New York State Department of Health, Albany, New York, USA (D.M. Lamson, M. Shudt, K. St. George);; Lovelace Respiratory Research Institute, Albuquerque, New Mexico, USA (A. Kajon);; Centre Hospitalier Universitaire Dr-Georges-L.-Dumont, Moncton, New Brunswick, Canada (G. Girouard)

**Keywords:** next-generation whole-genome sequencing, MiSeq, detection, genetic characterization, Adenoviridae, human adenovirus, HAdV, serotype 14, human adenovirus 14p1, viruses, influenza-like illness, differential diagnosis, New York, New Mexico, United States, Canada

## Abstract

During the 2014–15 influenza season, 13/168 respiratory samples from students with influenza-like illness (ILI) at a college in New York, USA, were positive for human adenovirus (HAdV); 4/13 samples were positive for HAdV-B14p1. During influenza season, HAdV should be included in the differential diagnostic panel used to determine the etiology of ILI.

Human adenoviruses (HAdVs) contain linear, double-stranded DNA with an average genomic length of 35 kbp. The 51 serotypes identified by seroneutralization and >70 genotypes that have been described by computational analysis of complete genomic sequences are classified within 7 species, designated HAdV-A through HAdV-G (*1*). HAdVs can cause a broad spectrum of disease, including gastroenteritis, conjunctivitis, upper and lower respiratory tract infections, hepatitis, and urinary tract infections (*2*). Most reported illnesses associated with HAdVs are caused by a limited number of types. HAdV type 14 of species HAdV-B (HAdV-B14), which was first identified in the Netherlands in 1955, was rarely reported until the emergence of a new genomic variant, 14p1, was documented in the United States in 2003 by military surveillance (*3*). In 2007 in the United States, sporadic outbreaks of HAdV-B14p1 infection occurred in Oregon (*4*) and at an air force base in Texas (*5*); subsequent outbreaks occurred in other locations in North America (*6*). The most striking genetic difference between variant 14p1 and the prototype strain deWit, which was isolated in the Netherlands in 1957, is a deletion in the fiber gene (*3*). Since its first detection in the United States, the 14p1 variant has been detected in association with acute respiratory diseases of variable severity in several states and countries (*5*,*7*–*10*).

A component of the influenza surveillance conducted by the New York State Department of Health includes the molecular testing and viral culture of respiratory samples submitted by sentinel physicians to the Wadsworth Center Virology Laboratory (Albany, NY, USA). Participating sentinel physicians collect samples from patients with influenza-like illness (ILI), which is defined as fever of >37.8°C plus cough or sore throat. During the 2014–15 influenza season, testing of surveillance specimens detected an increase in samples negative for influenza but positive for HAdV. We report results of sequence analysis for these HAdVs and compare severity of illness for patients infected with different virus types.

## The Study

During the 2014–15 influenza season, staff at the student health clinic of a college in Tomkins County, New York, collected respiratory samples from 168 students seeking care for ILIs and submitted them to the Wadsworth Center Virology Laboratory for testing. We tested all samples by conducting comprehensive respiratory virus culture and influenza virus detection (CDC Human Influenza Virus Real-time RT-PCR Diagnostic Panel; Centers for Disease Control and Prevention, Atlanta, GA, USA). For samples with HAdV-positive results, we performed confirmatory testing by using an adenovirus monoclonal antibody–specific immunofluorescence assay (Light Diagnostics Respiratory Panel Viral Screening and Identification IFA Kit; EMD Millipore, Billerica, MA, USA), and we conducted HAdV molecular typing by partial hexon and fiber gene sequence analysis (*11*,*12*). 

Of the 168 samples, 12 (7.8%) were positive for HAdV: HAdV-E4 (8 samples), HAdV-B14 (3 samples), or HAdV-C2 (1 sample), and 1 was suspected to be co-infected with HAdV-E4 and HAdV-B14. For further characterization, we obtained cultured isolates from 2 of the 3 HAdV-B14–positive samples and from the suspected HAdV-E4 and HAdV-B14 co-infected sample. We extracted intracellular genomic HAdV DNA as previously described (*13*) and used it for genome typing by restriction enzyme analysis and for whole-genome sequencing on the MiSeq platform (Illumina, Inc., San Diego, CA, USA). For genomic comparison by whole-genome sequencing, we also processed DNAs from previously characterized HAdV-B14 strains detected in the same geographic region as the study strains; 3 of the strains were isolated in 2011 in New Brunswick, Canada (*10*), and 1 was isolated in 2008 in New York (*3*). We uploaded the fully annotated whole-genome sequences from this study to GenBank (accession nos. KY201426–32).

**Figure 1:**
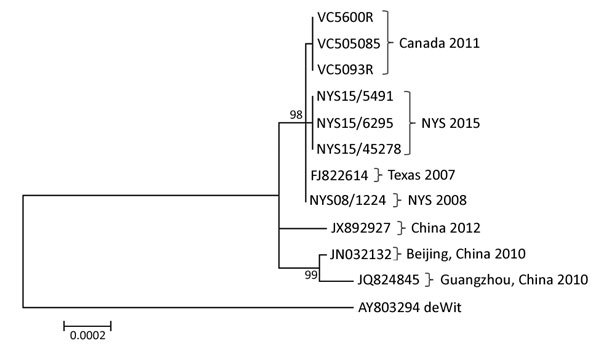
Phylogenetic tree of human adenoviruses constructed using 7 sequences obtained from college students with influenza-like illness, New York, USA, 2014–2015, and reference sequences of isolates from China (GenBank accession nos. JX892927, JN032132, and JQ824845); an isolate from Texas, USA (accession no. FJ822614); and the prototype strain, de Wit, from the Netherlands (accession no. AY803294). The tree was created by using the maximum-likelihood method based on the Kimura 2-parameter model with 500 bootstrap replicates from the whole-genome sequence of the displayed sequences. Pairwise distances were estimated by using the maximum composite–likelihood approach. All positions containing gaps and missing data were eliminated. Evolutionary analyses were conducted in MEGA6 (*14*). Scale bar indicates number of substitutions per site. NYS, New York State.

By in silico restriction enzyme analysis of their genomic sequence, we confirmed that 7 HAdV-B14 strains were genome type 14p1: of them, 3 were among the 4 strains isolated in New York during 2014, 1 was isolated in New York during 2008, and 3 were the strains isolated in Canada during 2011. The fourth 2014 HAdV-B14–positive specimen from New York did not grow in culture, but sequence analysis of the hexon gene and reactivity on a HAdV-B14–specific real-time PCR assay (*4*) identified the virus as HAdV-B14. We aligned and analyzed the 7 HAdV-B14 genomic sequences from this study along with 5 HAdV-B14 sequences available from GenBank (accession nos. AY803294, FJ822614, JN032132, JQ824845, and JX892927). The resulting tree (Figure 1) demonstrated that the HAdV-B14 strains from New York college students with ILI during the 2014–15 influenza season were closely related to strains circulating in North America during previous years, but they were distinguishable from strains isolated from 3 patients in 2011 in New Brunswick. The HAdV-B14 strains isolated from the New York students differed by 1–29 nt from the Canada 2011, Texas 2007, and New York 2008 strains and from strains isolated in China in 2010 and 2012; one aa change was observed in each of the following proteins: E1A, E1B, protein VI, and E3 20.8-kDa.

We did not perform restriction enzyme analysis on the isolate from the patient sample suspected to be co-infected with HAdV-E4 and HAdV-B14. However, virtual digest of the individual de novo–assembled, contiguous, whole-genome sequencing identified HAdV-E4 as genome type 4a1 and HAdV-B14 as 14p1 (data not shown).

**Figure 2:**
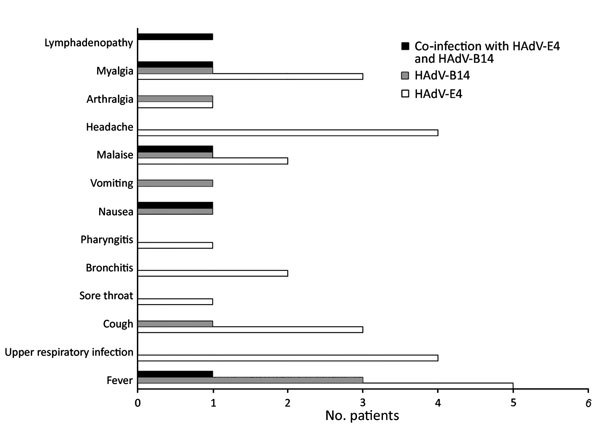
Symptoms experienced by 12 of 13 students with influenza-like illness who were found to be infected with human adenovirus (HAdV)-E4 (n = 8) or HAdV-B14 (n = 3) or co-infected with HAdV-E4 and HAdV-B14 (n = 1), New York, USA, 2014–2015.

Although we could not review patient charts, ILI symptoms and signs were noted on the original test request submission forms, which were available for our evaluation of New York case-patients (Figure 2). All 3 of the HAdV-B14–infected patients had fever, compared with only 5 of 8 HAdV-E4–infected patients (Figure 2). One of the HAdV14–positive patients had fever, nausea, vomiting, arthralgia, malaise, and myalgia; this combination of signs and symptoms was not noted for any other patients. However, the HAdV-C2–infected patient showed similar symptoms, along with bronchitis.

## Conclusions

Outbreaks of HAdV-associated febrile respiratory illness are common among nonvaccinated trainees in military recruit training centers (*15*). The military trainee environment and the college student setting share similarities, including population age (18–21 years) and multiple-occupant residences, which result in close human proximity and facilitate virus transmission. Thus, it is not surprising that HAdV infections are also prevalent among college students. Without active surveillance for HAdV and other respiratory pathogens, outbreaks of acute respiratory illness may go undetected, leading to rapid spread and, subsequently, to difficult containment.

Molecular testing is the primary laboratory surveillance tool. However, clinicians and laboratories in New York detected infections with HAdV and other respiratory pathogens by using viral culture on a portion of respiratory samples collected as part of the influenza surveillance program. During the 2014–15 influenza season, several samples submitted from colleges in New York tested positive for HAdV, but cases of HAdV-B14 infection were identified in only 1 college. Those infections may represent isolated cases, but during influenza season, clinicians and laboratories should be aware of HAdV as a possible cause of ILI and of the potential for HAdV-B14 to circulate at low frequency. This HAdV type has demonstrated potential to cause severe illness and has been responsible for widespread outbreaks (*3*–*6*,*8*,*9*). 

Our findings highlight the benefit of including college student populations in sentinel surveillance efforts. During influenza season, HAdV should be included in the differential diagnostic panel used to determine the etiology of acute respiratory disease to prevent the unnecessary use of influenza antiviral drugs. Furthermore, our findings demonstrate the power of next-generation sequencing for phylogenetic analysis of HAdV strains for investigation of outbreaks.
